# Using deep learning to shorten the acquisition time of brain MRI in acute ischemic stroke: Synthetic T2W images generated from b0 images

**DOI:** 10.1371/journal.pone.0316642

**Published:** 2025-01-06

**Authors:** Yun Peng, Chunmiao Wu, Ke Sun, Zihao Li, Liangxia Xiong, Xiaoyu Sun, Min Wan, Lianggeng Gong

**Affiliations:** 1 Department of Radiology, The Second Affiliated Hospital, Jiangxi Medical College, Nanchang University, Nanchang, Jiangxi, China; 2 Jiangxi Provincial Key Laboratory of Intelligent Medical Imaging, Nanchang, China; 3 Department of Electronic Information Engineering, Nanchang University, Nanchang, China; Al-Nahrain University, IRAQ

## Abstract

**Objective:**

This study aimed to assess the feasibility of the deep learning in generating T2 weighted (T2W) images from diffusion-weighted imaging b0 images.

**Materials and methods:**

This retrospective study included 53 patients who underwent head magnetic resonance imaging between September 1 and September 4, 2023. Each b0 image was matched with a corresponding T2-weighted image. A total of 954 pairs of images were divided into a training set with 763 pairs and a test set with 191 pairs. The Hybrid-Fusion Network (Hi-Net) and pix2pix algorithms were employed to synthesize T2W (sT2W) images from b0 images. The quality of the sT2W images was evaluated using three quantitative indicators: Peak Signal-to-Noise Ratio (PSNR), Structural Similarity (SSIM), and Normalized Mean Squared Error (NMSE). Subsequently, two radiologists were required to determine the authenticity of (s)T2W images and further scored the visual quality of sT2W images in the test set using a five-point Likert scale. The overall quality score, anatomical sharpness, tissue contrast and homogeneity were used to reflect the quality of the images at the level of overall and focal parts.

**Results:**

The indicators of pix2pix algorithm in test set were as follows: PSNR, 20.549±1.916; SSIM, 0.702±0.0864; NMSE, 0.239±0.150. The indicators of Hi-Net algorithm were as follows: PSNR, 20.646 ± 2.194; SSIM, 0.722 ± 0.0955; NMSE, 0.469 ± 0.124. Hi-Net performs better than pix2pix, so the sT2W images obtained by Hi-Net were used for radiologist assessment. The two readers accurately identified the nature of the images at rates of 69.90% and 71.20%, respectively. The synthetic images were falsely identified as real at rates of 57.6% and 57.1%, respectively. The overall quality score, sharpness, tissue contrast, and image homogeneity of the sT2Ws images ranged between 1.63 ± 0.79 and 4.45 ± 0.88. Specifically, the quality of the brain parenchyma, skull and scalp, and middle ear region was superior, while the quality of the orbit and paranasal sinus region was not good enough.

**Conclusion:**

The Hi-Net is able to generate sT2WIs from low-resolution b0 images, with a better performance than pix2pix. It can therefore help identify incidental lesion through providing additional information, and demonstrates the potential to shorten the acquisition time of brain MRI during acute ischemic stroke imaging.

## Introduction

Acute Ischemic stroke (AIS), one of the most common diseases in the elderly, accounts for 60% of strokes and has a high clinical mortality and disability rate [1–3]. Rapid and accurate diagnosis is closely related to the prognosis and the subsequent quality of life in AIS patients [4]. Owing to the high sensitivity of Magnetic Resonance (MR) imaging, particularly the Diffusion Weighted (DW) imaging, which is sensitive to the restricted diffusion of free water induced by cytotoxic edema in cerebral infarction areas, it has emerged as the most crucial imaging technique for AIS diagnosis [5,6]. Conventional brain MR scans typically encompass DW, T1 weighted (T1W), T2 weighted (T2W), and T2 FLAIR imaging sequences, all of which are time-consuming to acquire. Given the criticality of time in AIS, encapsulated in the phrase “time is life”, there may be a need for further optimization of these scanning procedures [7,8]. Shortening the acquisition time of brain MR imaging, such as reducing which of above sequences mentioned, will provide an important therapeutic time window for the disease.

Some medical institutions have begun to use a single DW imaging to rule out AIS, but single sequence imaging can lead to a lot of information loss, making it difficult to diagnose other lesions (e.g., hemorrhagic stroke) [9]. DWI scanning requires images with multiple b-values. Typically, the b = 0 sec/mm^2^ value image (b0 image) should be obtained first, followed by images with high b-value (e.g., 800, 3000 sec/mm^2^) [10]. Although the b0 image bears visual similarity to the T2W image, it is significantly less sharp than regular T2W images. The synthetic T2W (sT2W) images generated from the b0 images could shorten the acquisition time of brain MRI for stroke evaluation, and in the other hand, provide more information for the clinic to rule out other lesions, such as hemorrhage and otitis media.

Currently, there is burgeoning interest in utilizing deep learning algorithms to generate specific images, with the potential benefits of enabling immediate diagnosis, shortening scanning time, and reducing metabolic stress by minimizing the use of contrast agents [11,12]. Some studies proposed a novel invertible neural network for multimodal image translation [13]. Zhou et al. introduced the Hybrid-Fusion Network (Hi-Net) that comprehensively generates target modality images based on multimodality images, demonstrating advantages in MRI generation [14]. Specifically, the Hi-Net learns the mapping from the source image to the target image through three modules: the modality-specific network learns the representation of the input modality, and the fusion network learns its common latent representation, and then combines the latent representation with hierarchical features of the input modality through synthesis network to synthesize the target image.

Therefore, the aim of this study was to use Hi-Net to generate sT2W images from B0 images to explore the feasibility of shortening the acquisition time of brain MRI and excluding other lesions during the diagnosis of AIS.

## Materials and methods

### 1. Data collection

The Medical Research Ethics Committee of the hospital approved the study, and the requirement for written informed consent was waived. All data were fully anonymized during the analyses. The data for this study were accessed on February 9, 2024. This retrospectively study enrolled the individuals who underwent conventional brain MRI scans in the Hospital between September 1, 2023 and September 4, 2023. Exclusion criteria comprised poor image quality and age less than 18 years. Ultimately, the MRIs of 53 patients were included.

### 2. Image generation using Hi-Net

#### 2.1 Data preprocessing

To accelerate the convergence of the deep learning network, the input image is first normalized by the min-max normalization method. The calculation formula is as follows:

$$x_{new}=\frac{x-x_{\min}}{x_{\max}-x_{\min}}$$ (1)

Where x_new_ is the normalized image data, x_max_ is the maximum value of the input image data, and x_min_ is the minimum value of the input image data.

**Fig 1:**
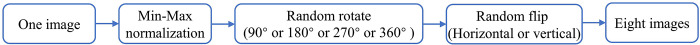
Data enhancement flow chart.

The performance of the model can be improved by using the data enhancement method. The original image of the training set is randomly rotated at specific angle (90°, 180°, 270°, or 360°). Following horizontal or vertical inversion, the new image is reshaped to 256×256. This process generates eight new images from each original image, thereby expanding the training set eightfold **(Fig 1)**.

#### 2.2 Image calculation

Hi-Net learns the mapping from existing images to target images for specific synthesis tasks. The model consists of three main components: Modality-Specific Network, Multi-Modal Fusion Network and Multi-Modal Synthesis Network [14].

**Fig 2:**
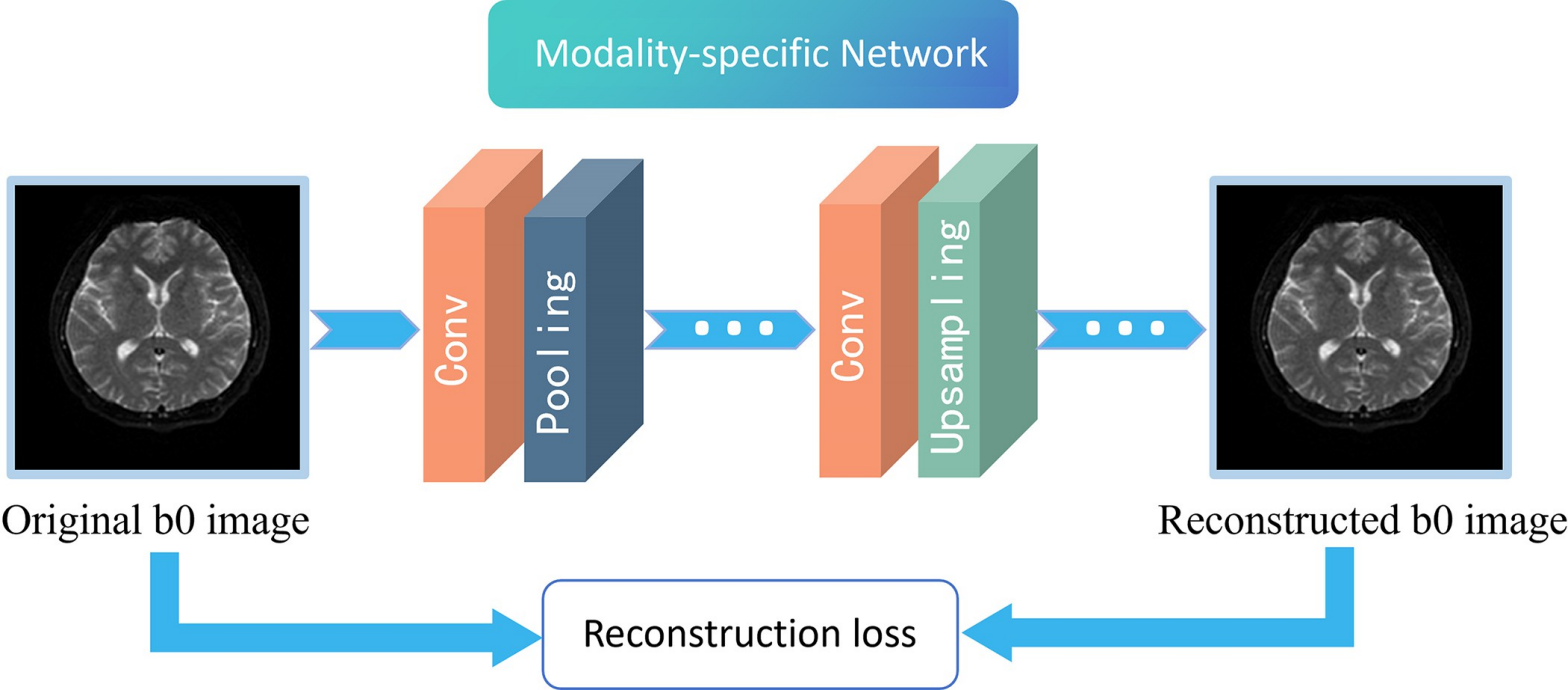
Workflow of modality-specific network. It illustrates how the original b0 image is reconstructed through a modality-specific network to capture the characteristic properties of the modality.

*A*. *Modality-Specific Network*. The modality-specific network can capture the unique property information of each modality and exploit the correlation between modalities to improve model learning performance. The loss function for this network is as follows:

$$L^{R}={\sum_{i}\left\|x_{i}-{\hat{x}}_{i}\right\|}_{1}$$ (2)

where *x*_*i*_ represents each individual modality and ${\hat{x}}_{i}$ denotes the reconstructed image of *x*_*i*_, ‖⋅‖_1_ represents the *L*_1_-norm. As shown in **Fig 2**, the network consists of convolutional layer, pooling layer, upsampling layer, and activation functions, which are used to reconstruct the b0 image.

*B*. *Multi-Modal Fusion network*. The multi-modal fusion network utilizes acquired low-level and high-level features to learn the similarities and differences among different modalities. It connects multiple modules to dynamically weight different fusion strategies, establishing connections for different modalities images.

*C*. *Multi-Modal Synthesis Network*. The design of multi-modal synthesis network is based on the principle of Generative Adversarial Networks (GAN), which consists of generator and discriminator. The key concept is to carry out continuous adversarial learning between generator and discriminator. The generator attempts to produce a T2W image that confuses the discriminator, while the discriminator endeavors to distinguish the generated T2W images from the real T2W images. Accordingly, the objective function of the generator can be formulated as:

$$\underset{G}{\min}\;\underset{D}{\max}V(D,G)=E_{x∼p_{data}(x)}[\log(D(x))]+E_{z∼p_{z}(z)}[\log(1-D(G(z)))]$$ (3)

where *x* represents the real image, *G*(*z*) represents the image generated by the G network, *D*() represents the probability of the D network judging whether the image is real. During the training process, the network D is trained to maximize log(*D*(*x*)) and log(1−*D*(*G*(*z*))), and the network G is trained to minimize log(1−*D*(*G*(*z*))), that is, to maximize the loss of D, making the generated samples more realistic.

**Fig 3:**
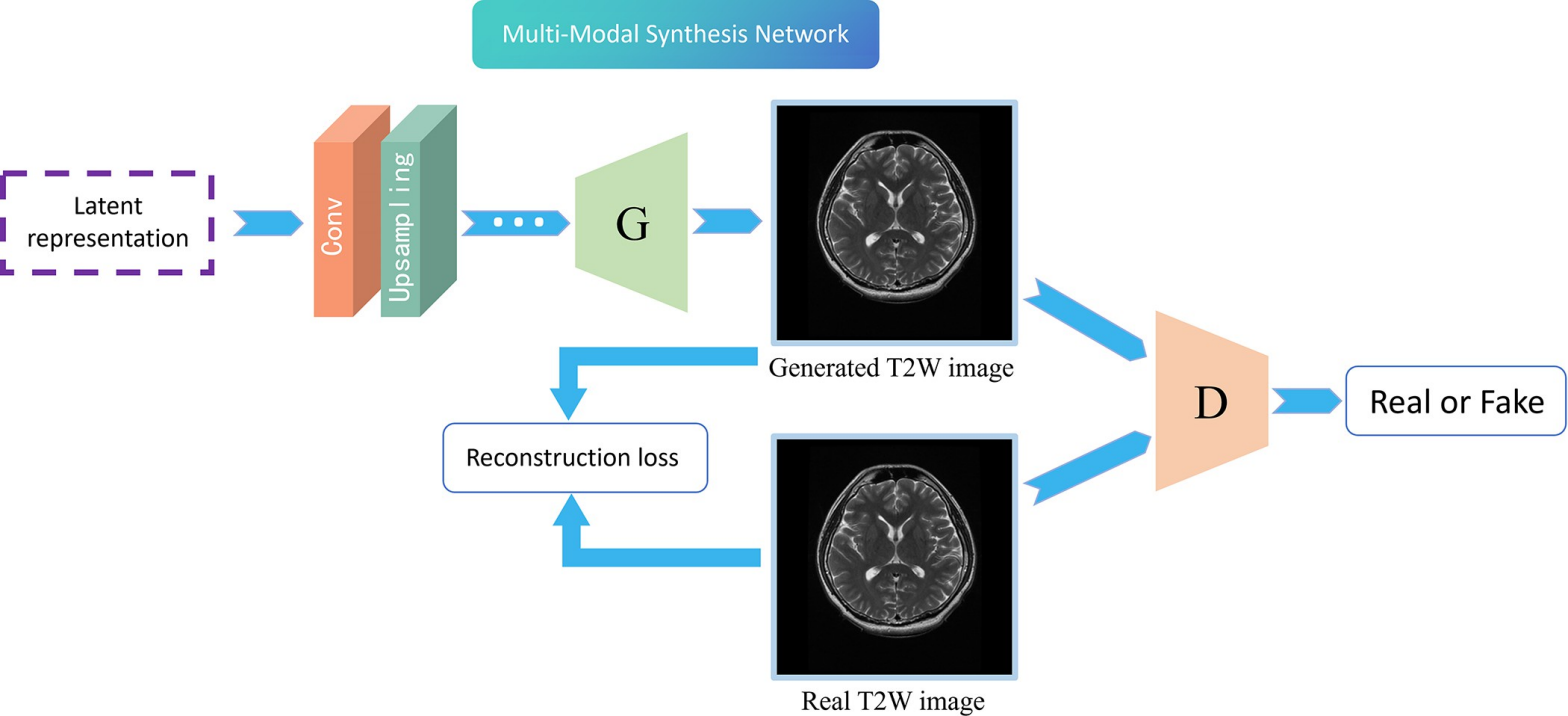
Workflow of the multi-modal synthesis network. The multi-modal latent representation generated by the last module in the multi-modal fusion network is synthesized into T2W image through the generator. The generated T2W image and the real T2W image are concurrently transmitted to the discriminator, and the gap between the generated image and the real image is reduced by the loss function.

As shown in **Fig 3**, during the training, the synthesis network calculating the loss between the generated image and the real image. The model is then trained using the weight of the loss adjusted model. The generated image gradually approaches the real image, rendering the discriminator incapable of distinguishing the generated image and the authentic image.

#### 2.3 Image generation using pix2pix

In order to better verify the effectiveness of the model, an advanced algorithm pix2pix were used to comparison [15]. The pix2pix GAN was the first success to use a conditional GAN to learn the mapping between paired images. It was designed for general purpose image-to-image translation [16].

All networks are trained using an ADAM trainer. A total of 4000 epochs were used to run the model, with an initial learning rate of 0.001. This code is implemented by the PyTorch library.

### 3. Image evaluation

#### 3.1 Quantitative image evaluation

The T2W image was utilized as the gold standard for the quantitative evaluate the sT2W image generated by the two algorithms. The evaluation metrics included the Peak Signal-to-Noise Ratio (PSNR), Structural Similarity (SSIM), and Normalized Mean Squared Error (NMSE).

The PSNR is generally used as an engineering parameter relating to the maximum signal and background noise. The PSNR is defined by

$$MSE=\frac{1}{H\times W}\sum_{i=1}^{H}\sum_{j=1}^{W}{\left(X(i,j)-Y(i,j)\right)}^{2}$$ (4)

$$PSNR=10\;\mathrm{lg}(\frac{{\left(2^{n}-1\right)}^{2}}{MSE})$$ (5)

Where, *H*, *W* are the height and width of the image *X* or *Y*, *MSE* represents the mean square error; and *n* is the number of bits per pixel. The unit of PSNR is usually dB.

The SSIM is a measure of structural similarity between the two images, which is defined as:

$$SSIM=\frac{\left(2\mu_{X}\mu_{Y}+C_{1})(2\sigma_{XY}+C_{2}\right)}{\left(\mu_{X}^{2}+\mu_{Y}^{2}+C_{1})(\sigma_{X}^{2}+\sigma_{Y}^{2}+C_{2}\right)}$$ (6)

Where *C*_1_ and *C*_2_ are constants to avoid the situation where the denominator is 0. *μ* represents the mean of image. *σ* represents the standard noise variance of image. *σ*_*XY*_ represents the covariance of image *X* and image *Y*.

The NMSE is calculated as:

$$NMSE(X,Y)={\left\|X-Y\right\|}_{2}^{2}/{\left\|X\right\|}_{2}^{2}$$ (7)

A higher PSNR, higher SSIM and lower NMSE indicate that the quality of the synthesized image is better and closer to the real image.

#### 3.2 Visual assessment

Two readers, Radiologist 1 (a junior radiologist with 5 years of experience) and Radiologist 2 (an attending junior radiologist with 10 years of experience), were required to determine the authenticity of (s)T2W images and further scored the visual quality of sT2W images in the test set using a five-point Likert scale. The relevant visual assessments were divided into two experiments.

In Experiment 1, the 191 pair of original T2W and sT2W images in the test set were randomly numbered as 001–382. Two readers were tasked with independently evaluating the properties of the images (real or synthetic) one by one without knowing the nature of the image.

In Experiment 2, the readers have been told of the authenticity of the T2W images. They were required to further rate the quality of the sT2W images relative to the real images. The quality of overall synthetic image and focal region were both assessed by employing a 5-point Likert scale (5 = very good, 4 = good, 3 = acceptable, 2 = poor, 1 = very poor). Four metrics: overall image quality, sharpness, tissue contrast, and image homogeneity were scored to indicate the overall quality of each sT2w image [15]. Subsequently, three metrics: the sharpness, tissue contrast, and image homogeneity were scored to indicate the quality of focal area. The focal parts included the brain parenchyma, skull and scalp, orbits, middle ear, and paranasal sinuses. This study defined sharpness as the clarity of anatomical structures among the brain parenchyma, cerebrospinal fluid, and skull. Tissue contrast refers to the contrast between the above tissues, and image homogeneity refers to the uniformity and stability of the image.

### 4. Statistical analysis

All the statistical analysis were performed using SPSS software (version 26; IBM, Armonk, NY, USA). All evaluation values, including quantitative parameters and Likert scores, are expressed as the mean ± standard deviation.

The weighted kappa coefficient was used to evaluate the consistency of the image quality scores between the two observers. The degrees of consistency were as follows: < 0.20, slight; 0.21–0.40, fair; 0.41–0.60, moderate; 0.61–0.80, substantial; and 0.81–1.0, almost perfect.

## Results

### 1. Basic data

The MRI of 53 patients were ultimately included, with each patient contributing 18 pairs of 2D T2W and b0 images. A total of 954 pairs of images were divided into the training set (763 pairs) and the test set (191 pairs).

### 2. Quantitative assessment of images

**Fig 4:**
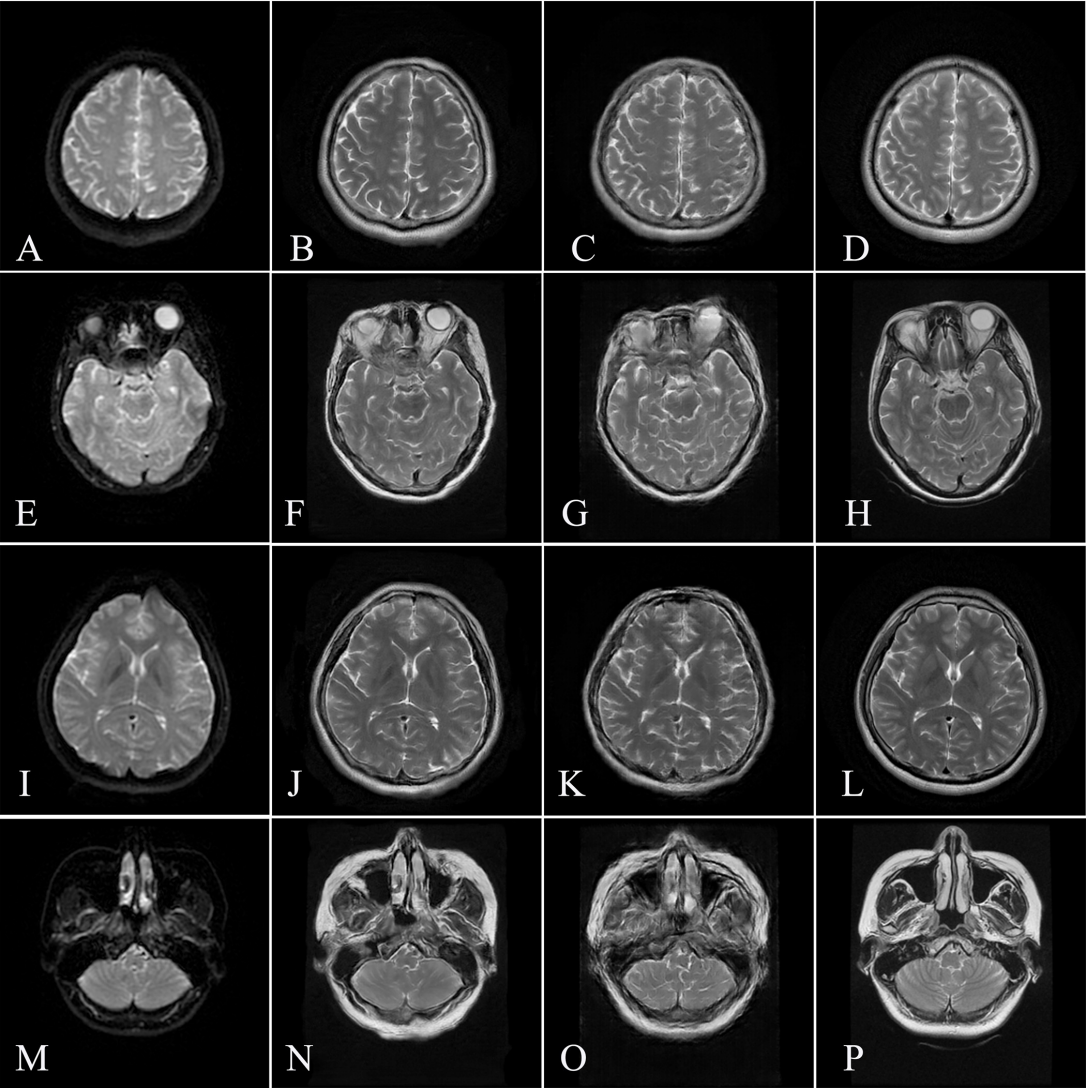
Representative image in the test set. The left column shows the DWI b0 image (A/E/I/M), the middle column shows the sT2WI images generated by Hi-Net (B/F/J/N), and pix2pix (C/G/K/O) the respectively, and the right column shows the original T2WI images (D/H/L/P).

**Fig 4** shows a schematic diagram of the images from test set. In the quantitative evaluation of images generated by Hi-Net in the test set, the parameters were as follows: PSNR, 20.646 ± 2.194; SSIM, 0.722 ± 0.0955; NMSE, 0.469 ± 0.124. The parameters of images generated by pix2pix were as follows: PSNR, 20.549 ± 1.916; SSIM, 0.702 ± 0.0864; NMSE, 0.239±0.150. Hi-Net algorithm presented higher PSNR, SSIM and lower NMSE than pix2pix, indicated that Hi-Net performs better.

### 3. Visual assessment

#### 3.1 The images generated by Hi-Net were further analyzed

In Experiment 1, **Table 1** shows the authenticity judgments by reader 1 and reader 2. The percentages of correct identification of image nature by the two readers were 69.90% (267/382) and 71.20% (272/382), respectively. The percentages of false identification of synthetic image as real were 57.6% (110/192) and 57.1% (109/192), respectively.

**Table 1:** Judgment of the authenticity of the images by two radiologists.

	Reader 1		Reader 2	
	True	Synthetic	True	Synthetic
T2W images (n = 191)	186	5	190	1
Synthetic T2W images by Hi-Net (n = 191)	110	81	109	82

#### 3.2 In Experiment 2, the scores of the sT2W images are shown in Table 2

The average kappa coefficient between the two radiologists for evaluating image quality was 0.655 (range, 0.498–0.774; P < 0.001) for the overall evaluation and 0.468 (range, 0.270–0.662; P < 0.001) for the focal regions. The overall image quality, anatomical sharpness, tissue contrast and image homogeneity of sT2W images were rated as good to excellent. The scores for different focal areas suggested that the image quality for the middle ear was very good, for the brain parenchyma, skull, and scalp was good. The image quality for the orbit and paranasal sinus regions was poor to acceptable.

**Table 2:** Evaluation of the overall and focal quality of the images.

	Reader 1	Reader 2	Kappa	P value
**Overall**				
Overall image quality	3.43 ± 1.23	3.51 ± 1.29	0.774	<0.001
Sharpness	3.82 ± 1.10	3.35 ± 1.08	0.749	
Tissue contrast	4.05 ± 1.08	3.70 ± 0.99	0.498	
Image homogeneity	3.55 ± 0.97	3.50 ± 1.00	0.597	
**Focal regions**				
**Brain parenchyma**				
Sharpness	4.08 ± 1.02	3.87 ± 0.91	0.529	<0.001
Tissue contrast	4.34 ± 0.92	4.17 ± 0.83	0.507	
Image homogeneity	3.93 ± 1.01	3.87 ± 0.90	0.525	
**Skull and scalp**				
Sharpness	3.89 ± 1.32	3.79 ± 1.03	0.434	<0.001
Tissue contrast	4.07 ± 1.25	4.03 ± 0.94	0.394	
Image homogeneity	3.16 ± 1.13	3.63 ± 1.01	0.424	
**Orbit**				
Sharpness	2.05 ± 0.84	2.21 ± 1.12	0.610	<0.001
Tissue contrast	2.33 ± 1.19	2.37 ± 1.31	0.662	
Image homogeneity	1.84 ± 0.92	2.21 ± 1.21	0.572	
**Middle ear**				
Sharpness	4.00 ± 0.94	4.09 ± 0.94	0.569	<0.001
Tissue contrast	4.45 ± 0.88	4.30 ± 0.90	0.475	
Image homogeneity	4.25 ± 0.99	4.02 ± 0.93	0.445	
**Paranasal sinuses**				
Sharpness	2.05 ± 0.98	1.63 ± 0.81	0.273	<0.001
Tissue contrast	2.19 ± 1.16	1.91 ± 1.00	0.328	
Image homogeneity	1.92 ± 1.02	1.63 ± 0.79	0.270	

## Discussion

To the best of our knowledge, this is the first preliminary exploration proposing the generation of high-resolution T2W images based on DWI b0 images, with the aim of shortening the acquisition time of brain MRI in AIS. We employed the Hi-Net and pix2pix algorithm to synthesize T2W images. Hi-Net algorithm presented higher PSNR, SSIM and lower NMSE than pix2pix, indicated that Hi-Net performs better. When compared with conventional T2W images, sT2W images generated by Hi-Net achieved acceptable or even excellent in terms of quantitative parameters and radiologists’ visual scores.

To generate translated modality images from given modality images is one of the popular directions in deep learning. To minimize the use of contrast agents, Müller-Franzes et al. utilized GANs to explore the synthesis of enhanced images from T1 combined with T2 images or low-contrast agent images [17]. Fujita et al. develop a deep learning algorithm to generate MR angiography images based on 3D synthetic MRI raw data, which may be useful as a screening tool for intracranial aneurysms without requiring additional scanning time [18]. To reduce radiation dosimetry, an increasing number of researchers using deep learning on head and neck MR images to generate corresponding CT images [13,19,20]. Image generation across modalities can aid in simplifying clinical examination procedures and reducing unnecessary expenditures. We hope to use deep learning algorithm to generate sT2W images to avoid T2W image scanning and speed up brain MR imaging acquisition during stroke screening.

Compared with b0 image, the quality of sT2W image by the two algorithms, Hi-Net and pix2pix, is improved. Hi-Net algorithm presented higher PSNR, SSIM and lower NMSE than pix2pix, indicated that Hi-Net performs better. Currently, prevalent image generation algorithms are rooted in the Variational Autoencoder (VAE), PixelRNN/PixelCNN, and GAN series networks. The VAE network, characterized by its robust reasoning properties, is commonly used for simple digital fitting and image completion [21,22]. It can be applied to AI face modification and beauty cameras, but is not good at generating clear images [23]. The PixelRNN/PixelCNN model utilizes the probability chain rule and generates images pixel by pixel. The advantage of this model is its high-quality images, typically widely applied to image inpainting and coloring [24]. However, the speed of image generation by this network is very slow in practical application [24]. GAN produces relatively high quality images, and the dynamic interplay between the generator and the discriminator make it suitable for image enhancement [25]. However, some shortcoming such as training instability, pattern collapse and gradient disappearance limit its application. Therefore, various variants, such as conditional GANs and deep convolutional GANs, have emerged. Deep convolutional GAN is mainly used to improve the visual quality of generated image. The advantage of this approach is that the training is stable [26], but the model needs to readjust the parameters when training different data sets and is prone to model crash. Conditional GAN is mainly used for image completion, style transfer, and image subtitle generation [27,28], it implements conditional image generation by introducing conditional information into generator and discriminator. Pix2pix is based on conditional GAN and performs well on image generation and image conversion tasks [29–31]. Therefore, we choose pix2pix as the comparison algorithm, and finally find that the synthetic image obtained by this algorithm has lower quality than Hi-Net.

The Hi-Net model in this study uses existing modality data as input to synthesize the target modality. The modality-specific network is capable of capturing image feature information from a single modality image, while the fusion network discerns connections between different modalities. Furthermore, the synthetic network employs a generator and a discriminator to synthesize target modality, in conjunction with the correlation between the acquired features and modalities. Ultimately, the cross-domain generation of images is achieved by combining three models.

The visual assessment results from two radiologists indicated that the overall image quality generated by Hi-Net ranged from acceptable to excellent, with the display of the brain parenchyma, skull and scalp, and middle ear region being particularly remarkable, even similar to that of the original T2W images. We also observed that the quality of the regions close to the skull (especially paranasal sinus) and orbit were poorer than that the other regions. This phenomenon may be attributed to the fact that the signal acquisition of the skull base and orbital region is not the focal point in the process of DW image scanning, resulting in blurred or deformed appearance in these regions of the b0 image. Deep learning algorithms can generate images based solely on a limited number of signals. Nevertheless, we believe that, even with the current results, it is feasible to evaluate some crucial areas such as the brain parenchyma and middle ear using sT2W images.

This study has several limitations. Firstly, it constitutes an initial exploration based on a small sample size; multicenter studies with larger sample sizes are necessary to validate the results of this study. Secondly, although our experiments confirmed that the superior quality of the brain parenchyma, middle ear, skull, and scalp regions in the generated synthetic images, the quality of the orbit and paranasal sinus regions still requires further improvement. Subsequent studies will demand additional algorithms and larger sample sizes. Third, only the feasibility of T2W image generation from b0 image was explored, and the feasibility of deep learning to generated other sequence images, such as T1W, T2FLAIR, etc., based on DW images needs to be further studied.

## Conclusion

Deep learning algorithms including Hi-Net and pix2pix can generate high-resolution T2W images from low-resolution b0 DW images, and the former shows a higher quantitative index. The sT2W images generated by Hi-Net are acceptable when compared with the real T2W images. They could provide a clearer contrast and offer a significant advantage in revealing craniocerebral anatomy, especially in the middle ear, brain parenchyma, and skull regions. This finding suggests that it is feasible to use deep learning to generate T2W images from b0 images thereby assisting incidental lesion through providing additional information as well as reducing scanning time of brain MR imaging with multi-sequence during the AIS imaging.

## Abbreviations

AIS: Acute Ischemic stroke
MR: Magnetic Resonance
DW: Diffusion Weighted
T1W: T1 weighted
T2W: T2 weighted
b0 image: the b = 0 sec/mm^2^ value image
sT2W: synthetic T2W
Hi-Net: Hybrid-Fusion Network
GAN: Generative Adversarial Networks
PSNR: Peak Signal-to-Noise Ratio
SSIM: Structural Similarity
NMSE: Normalized Mean Squared Error
